# A novel fatty acid metabolism-related signature identifies MUC4 as a novel therapy target for esophageal squamous cell carcinoma

**DOI:** 10.1038/s41598-024-62917-z

**Published:** 2024-05-30

**Authors:** Shanshan Li, Zhengcao Liu, Qingqing Chen, Yuetong Chen, Shengjun Ji

**Affiliations:** 1 Department of Operating Room, Weifang Traditional Chinese Hospital, Weifang, China; 2 Department of Radiotherapy & Oncology, The Affiliated Suzhou Hospital of Nanjing Medical University, Gusu School, Nanjing Medical University, No.16 Baita Road, Suzhou, 215001 China

**Keywords:** Fatty acid metabolism, Esophageal squamous cell carcinoma, Prognosis, Immune microenvironment, Cancer, Computational biology and bioinformatics, Biomarkers, Molecular medicine, Oncology, Risk factors

## Abstract

Fatty acid metabolism has been identified as an emerging hallmark of cancer, which was closely associated with cancer prognosis. Whether fatty acid metabolism-related genes (FMGs) signature play a more crucial role in biological behavior of esophageal squamous cell carcinoma (ESCC) prognosis remains unknown. Thus, we aimed to identify a reliable FMGs signature for assisting treatment decisions and prognosis evaluation of ESCC. In the present study, we conducted consensus clustering analysis on 259 publicly available ESCC samples. The clinical information was downloaded from The Cancer Genome Atlas (TCGA, 80 ESCC samples) and Gene Expression Omnibus (GEO) database (GSE53625, 179 ESCC samples). A consensus clustering arithmetic was used to determine the FMGs molecular subtypes, and survival outcomes and immune features were evaluated among the different subtypes. Kaplan–Meier analysis and the receiver operating characteristic (ROC) was applied to evaluate the reliability of the risk model in training cohort, validation cohort and all cohorts. A nomogram to predict patients’ 1-year, 3-year and 5-year survival rate was also studied. Finally, CCK-8 assay, wound healing assay, and transwell assay were implemented to evaluate the inherent mechanisms of FMGs for tumorigenesis in ESCC. Two subtypes were identified by consensus clustering, of which cluster 2 is preferentially associated with poor prognosis, lower immune cell infiltration. A fatty acid (FA) metabolism-related risk model containing eight genes (FZD10, TACSTD2, MUC4, PDLIM1, PRSS12, BAALC, DNAJA2 and ALOX12B) was established. High-risk group patients displayed worse survival, higher stromal, immune and ESTIMATE scores than in the low-risk group. Moreover, a nomogram revealed good predictive ability of clinical outcomes in ESCC patients. The results of qRT-PCR analysis revealed that the MUC4 and BAALC had high expression level, and FZD10, PDLIM1, TACSTD2, ALOX12B had low expression level in ESCC cells. In vitro, silencing MUC4 remarkably inhibited ESCC cell proliferation, invasion and migration. Our study fills the gap of FMGs signature in predicting the prognosis of ESCC patients. These findings revealed that cluster subtypes and risk model of FMGs had effects on survival prediction, and were expected to be the potential promising targets for ESCC.

## Introduction

Esophageal cancer has an increasingly threat to human public health, with an approximate 604,000 new cases and 544,000 deaths by 2020 worldwide^1^. Esophageal squamous cell carcinoma (ESCC) accounts for more than 90% of pathological type in esophageal cancer^2,3^. Despite the emerging breakthroughs employed in the management of ESCC, intrinsic or acquired resistance of immunotherapy are still the main obstacle to the prognosis of ESCC patients, with a 5-years overall survival rate approximately 20%^4^. Recently, some studied conducted the ferroptosis-related genes signature^5^, hypoxia-related genes signature^6^, and autophagy-related genes signatures^7^ to evaluate the predictive value in ESCC patients, while the heterogeneous biological characteristics of patients makes it dissatisfied to precisely evaluate the prognosis of patient. Hence, it is imperative to establish reliable predictors to identify risk stratification for the individualized management of ESCC patients.

Rapid cell proliferation, growth, and dissemination increase alterations in metabolism, result in cancer cells metabolic reprogramming^8^. The increasing evidence supports that metabolic reprogramming exhibits a closely link with human cancer^9,10^. Metabolic heterogeneity is commonly found in different malignant tumors, which contributes to the difference of the curative effect of metabolic drugs and may results in drugs resistance^11,12^. Fatty acid (FA) metabolism, especially in uptake and synthesis of fatty acid (FAs), constitutes an important aspect of this reprogramming. FA metabolism, including FA uptake, synthesis, oxidation, and modification, has attracted increased research attention^13^. Accumulating evidence demonstrates that FAs are fundamental components of cancer cells, and FAs are essential for store energy, cell membrane proliferation, and producing signaling molecules (such as PI3K/Akt/mTOR)^14–16^. Metabolic rearrangements in cancer cells often exhibit the features of hyperactive FA synthesis and/or β-oxidation. Previous studies have shown that the inhibitions of enzymes of FA synthesis or beta-oxidation could inhibit the cancer cells development, proliferation and spread, which suggested that FA metabolism confers significant effects on tumor survival^17^. Notably, cancer cells can release FAs to weaken the anti-tumor immune activity of T-lymphocyte cells by inhibiting T-lymphocyte proliferation, cytokine production^18^. Additionally, promoting FA catabolism can remarkably improve the efficacy of immunotherapy^19^. However, limited number of studies on FA metabolism-related genes signature (FMGs) has been investigated in ESCC.

In the present study, by combining the transcription profiles data, we systematically evaluated the prognostic value of cluster subtypes and risk model based on FMGs signature. We systematically analyzed the relationship among the FMGs signature and immune features in ESCC patients. Furthermore, we explored the inherent mechanisms of the target gene for tumorigenesis in vitro. Our study may provide new insights on FA metabolism for prognosis and tumor immune microenvironment (TME) heterogenicity for ESCC.

## Material and methods

### ESCC dataset acquisition

The clinical data and gene expression profile of 259 ESCC samples were obtained from The Cancer Genome Atlas (TCGA) and Gene Expression Omnibus (GEO) databases. Of all the ESCC samples, the RNA-seq data (FPKM format), regarding 80 ESCC samples, was retrieved for TCGA database. Meanwhile, GSE53625 incorporating gene expression profiles based on GPL18109 platform was downloaded from GEO database. The ‘caret’ package in R software randomly allocated all the ESCC samples (n = 259) into the training and testing cohorts in a 1:1 ratio. Subsequently, 130 ESCC samples were used as a training cohort, and 129 ESCC samples employing as the testing cohort.

We acquired the FA metabolism-related gene sets (KEGG fatty acid metabolism pathways, Reactome fatty acid metabolism genes, and Hallmark fatty acid metabolism genes^20–23^), and further analyzed for FMGs. FMGs were extracted from the Molecular Signature Database v7.4 (MSigDB).

### Identification of FMGs subtypes

The consensus clustering arithmetic is used to assess the cancer features differences within a specified data set^24^. To identify cluster subtypes of ESCC for optimization classification purposes in FMGs, consensus clustering was performed in all ESCC samples through the “ConsensusClusterPlus” package by the K-means algorithm, which comprised consensus matrix, cumulative distribution function (CDF), and delta area plots. Stable consensus clustering classification was presented with the specific parameters (re-samplings, 50; pltem, 0.8; pearson correlation distances, 1). The survival analysis between different cluster subtypes were performed through the Kaplan–Meier survival curves method, and chi-squared test was applied to explore the survival difference comparisons among different subtypes.

### Construction and validation of risk model

Based on the genes expression profiles and prognostic information intersected in training cohort, the FMGs that exhibited significance (*p* < 0.05) in univariate Cox regression analysis for survival were screen as the prognostic-related FMGs and then included into the least absolute shrinkage and selection operator (LASSO) analysis with tenfold cross-validation. Subsequently, LASSO analysis was applied for selecting best candidate FMGs into the risk model by using the “glmnet” R package. The specific risk score of each ESCC patients was analyzed based on the FMGs expression levels and corresponding regression coefficient, and the detailed formula was as follows: risk score = $\sum_{1}^{i}($gene Expression × gene coefficient). Subgroups including high-risk and low-risk groups were determined based on their respective median risk score. The same algorithm analysis was used for the testing cohort and all ESCC cohorts. The relationship between the risk score and the clinicopathological features (age, gender, T stage, N stage, and TNM stage) was explored. Additionally, the "survminer" and "timeROC" R packages were utilize to estimate overall survival (OS) difference and the predictive accuracy of the risk model, respectively.

### Nomogram construction

Clinicopathological variables and risk score were performed univariate and multivariate Cox regression analyses, and the “forestplot” R package was utilized to screen the independent prognostic factors. A nomogram was generated based on age, gender, T stage, N stage, TNM stage, and the risk score with “regplot”, “survival” and “rms” R packages. In this nomogram arithmetic system, each clinicopathological variable was serve as a score, and overall scores of all variables in each sample were calculated and exhibited. ROC curve was analyzed to evaluate the discrimination reliability of the nomogram.

### Functional enrichment analysis

Gene Ontology (GO) and Kyoto Encyclopedia of Genes and Genomes (KEGG, https://www.kegg.jp/kegg/kegg1.html) analyses of differentially expressed FMGs among different subtypes were conducted through the “org.Hs.eg.db” and “clusterProfiler” R packages employing all ESCC samples data. In addition, to further search inherent mechanisms associated with FMGs among the two risk groups, the gene set enrichment analysis (GSEA) was performed to distinguish hallmark pathways associated with FMGs using the “limma” R package, with c5.go.symbols.gmt and c2.cp.kegg.v7.0.symbols.gmt as reference gene sets. *p* < 0.05 and q false-discovery rate (FDR) value less than 0.25 were considered valid.

### Tumor microenvironment analysis

The composition of the 21 kinds of infiltrating immune cells and immune function were performed in ESCC samples using “CIBERSORT” R package, and the heatmap was visualized. The ESTIMATE algorithm identifies tumor microenvironment features associated with immune, stromal, and ESTIMATE score for each patient using “estimate” R package. The associations among the cluster subtypes, risk score, and immune checkpoint expression were detected using Spearman’s correlations. Kruskal Wallis test was applied to perform the differences analysis between cluster subtypes and risk groups.

### Quantitative real-time polymerase chain reaction (qRT-PCR)

Normal human esophageal epithelial cells (HEEC) and human ESCC cell lines ECA109 and TE1 were obtained by Scientific Research Center of the Fourth Hospital of Hebei Medical University. These cells were cultured with RPMI-1640 containing 10% Fetal Bovine Serum (FBS), at 37 °C with 5% carbon dioxide incubator. Total RNA of cells was isolated with TRIzol reagent (Thermo Fisher Scientific) and reverse-transcribed to cDNA with PrimeScript Strand cDNA Synthesis Kit (Thermo Fisher Scientific). qRT-PCR analysis was performed with MonAmp™ SYBR® Green qPCR Mix, and the risk model-relevant genes was analyzed with 2^−ΔΔCT^ method. The primer sequences of these gene and GAPDH are displayed in Table 1.

**Table 1:** The primer sequences of eight FMGs used for qRT-PCR (Sequence, 5ʹ– > 3ʹ).

Genes	Forward primer	Reverse primer
FZD10	AGCCATCCAGTTGCACGAG	GAGTCGGGCCACTTGAAGTT
TACSTD2	ACAACGATGGCCTCTACGAC	GTCCAGGTCTGAGTGGTTGAA
MUC4	CGTTCTGGGACGATGCTGAC	GATGGCTTGGTAGGTGTTGCT
PDLIM1	CCCAGCAGATAGACCTCCAG	TCTGAGCTTCCAAGTGTGTCATA
PRSS12	GGACAGCGCCACAACTTTTG	CGAAGTCGTACTGATCCGTGT
BAALC	GAGCCCCGCTACTACGAGA	AGTCGGTGTAGGTGAGCCA
DNAJA2	GTGGCTGACACGAAGCTGTA	AAGACCTTGCTCTCCGTATCT
ALOX12B	CCATCTCACTGACCATTGTGG	CAGGCGGATGATGATGAGC

*FMGs* Fatty acid metabolism-related genes.

### Cell culture

Human ESCC cell lines and HEEC cells, were obtained from the Research Center of the Fourth Hospital of Hebei Medical University (Shijiazhuang, China). For the current study, all cells were cultured in RPM1-1640 medium (Gibco, USA) containing 10% fetal bovine serum (FBS) (Invitrogen, USA) and 100 U/ml penicillin (Invitrogen, USA) and 100 μg/ml streptomycin (Invitrogen, USA) in the 37 °C and 5% CO_2_ cell incubator.

### Cell transfection

The ECA109 and TE1 cells were transfected with human MUC4 small interfering RNA (si-MUC4) and negative control shRNA (si-NC) using Lipofectamine 2000 (Invitrogen, USA) in accordance with the instructions of manufacturer. After 24 h, ECA109 and TE1 cells were harvested for subsequent functional assays.

### Proliferation assays

Cell proliferation was analyzed using Cell Counting Kit-8 (Med Chem Express Princeton, USA). ESCC cell lines were harvested and seeded into 96-well plates with five replicate wells for each group per experiment. Different groups of ESCC cells lines were incubated with 10 µl CCK-8 at 24 h, 48 h and 72 h. Then, absorbance was measured following the manufacturer’s instructions, the optical density (OD) value was determined at a wavelength of 450 nm by a Multiskan microplate (ELx800; Bio-Tek, Winooski, VT, USA). At least three times dependent experiments were repeated.

### Wound healing assay

The ECA109 and TE1 cells were plated in 6-well plates. When these ESCC cells grew to confluence > 80%, a straight cell-free zone was scratched using a 200-µl pipette tip. These cells were cultured in 2 ml of serum-free medium. The scratched areas were examined at 0 h, 24 h by using the microscope and photographed.

### Cell invasion assays

Transwells assays was used for testing the invasive ability of ESCC cells. ECA109 and TE1 cells were diluted with serum-free medium and 200 µl RPMI-1640 medium were seeded into the Matrigel-coated (Corning Inc. USA) upper chamber. 600 µl RPMI-1640 medium containing 20% FBS was added into the lower chamber. After incubation for 24 h with 37 °C constant temperature, cells on the upper side of the chamber were wiped off with cotton swabs, and the cells were fixed with 4% paraformaldehyde and stained with 0.1% crystal violet for 15 min. Representative images were captured under fluorescence microscope (Nikon Ti2, Japan).

## Results

### Identification of prognostic FMGs

**Figure 1:**
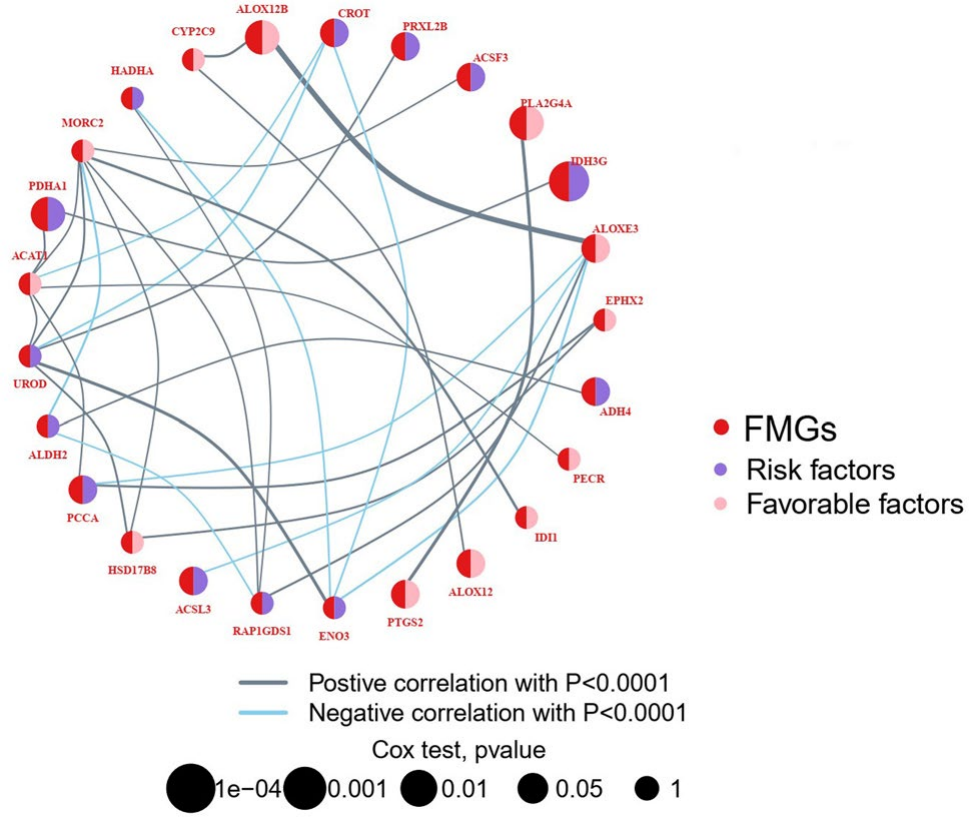
The FMGs interaction network of in ESCC patients. *FMGs* fatty acid metabolism-related genes, *ESCC* esophageal squamous cell carcinoma.

In this study, we identified prognostic-related FMGs among 259 ESCC samples in the all cohorts. The univariate Cox regression analysis identified 128 FMGs associated with prognosis. Moreover, the interaction correlations and degree of risk for prognosis of top thirty prognosis-related FMGs were illustrated by a network diagram (Fig. 1). The results indicated that PTGS2, ALOX12, IDI1, PECR, EPHX2, ALOXE3, PLA2G4A, ALOX12B, CYP2C9, MORC2, ACAT1, and HSD17B8 were favorable factors for OS, others were risk factors for OS.

### Establishment of tumor cluster subtypes

**Figure 2:**
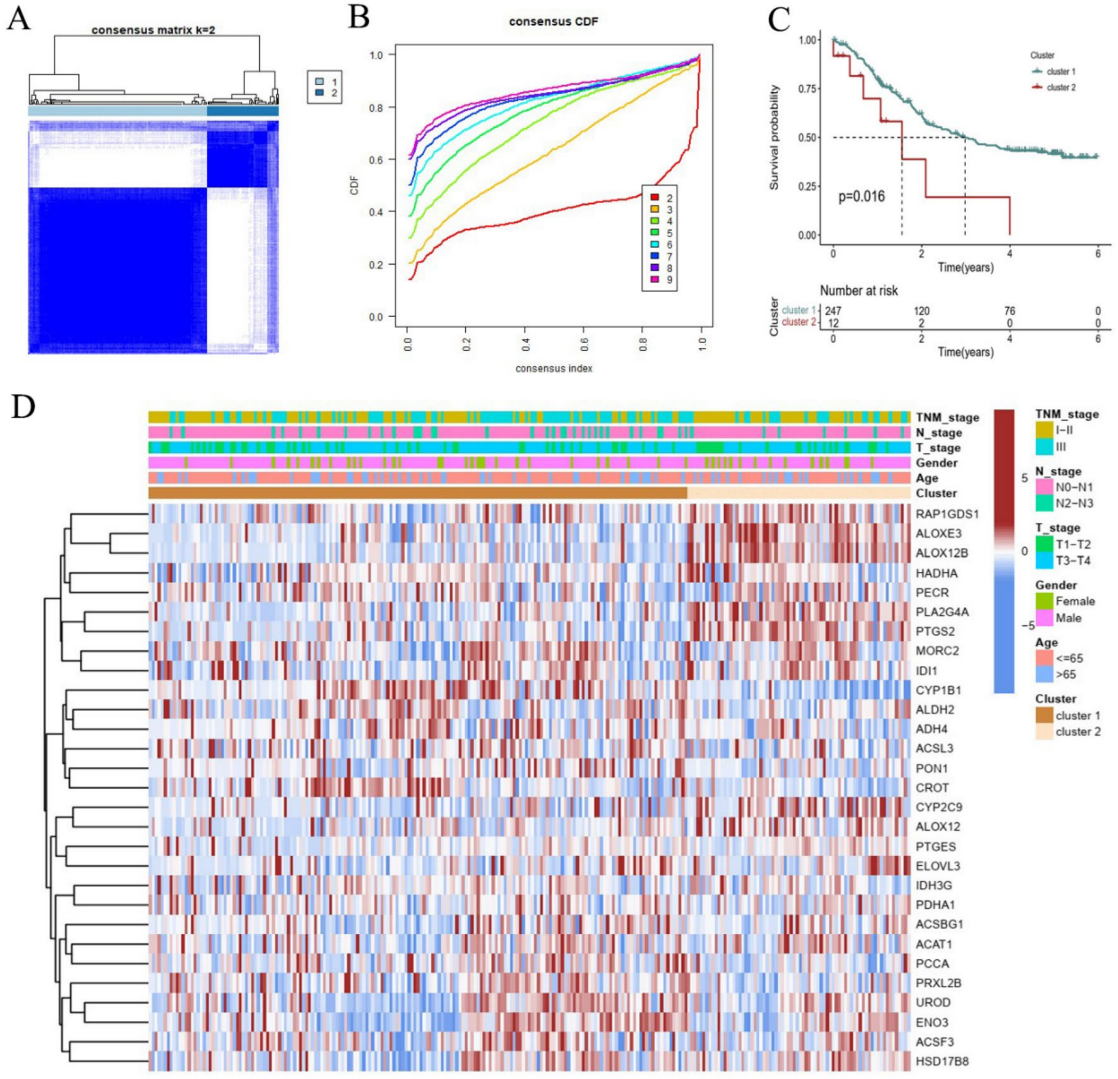
ESCC subtypes based on consensus clustering. (**A**, **B**) The ESCC patients were divided into cluster 1 and cluster 2 based on the prognostic FMGs. (**C**) The Kaplan–Meier analysis of the patients among two clusters. (**D**) A heatmap regarding to the relationships between the clinicopathological features and two clusters. *ESCC* esophageal squamous cell carcinoma, *FMGs* fatty acid metabolism-related genes.

Based on the screened prognosis-related FMGs expression data, the consensus matrix k value of 2, 259 ESCC patients were clustered into two subtypes (cluster 1 and cluster 2) with optimal consistency and stability (Fig. 2A,B). Kaplan–Meier survival analysis revealed that cluster 1 patients had a significantly poorer prognosis that in in cluster 2 patients (P = 0.0016; Fig. 2C). A heatmap depicted the prominent difference of prognosis-related FMGs expression and clinicopathological variables in these two clusters (Fig. 2D). These findings indicated that cluster subtypes could distinguish survival risk stratification of ESCC.

### Functional enrichment and immunological characterization of cluster subtypes

**Figure 3:**
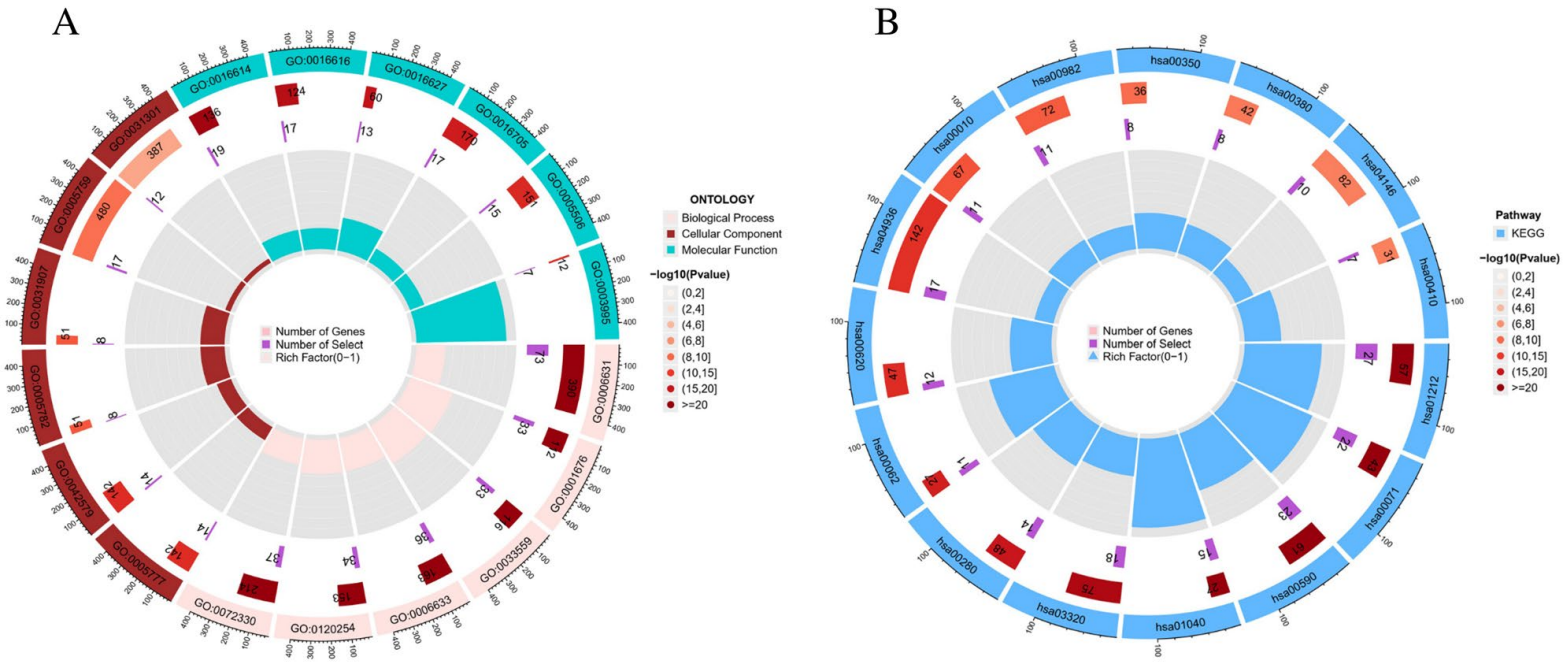
Functional enrichment analysis in two ESCC subtypes. (**A**) GO enrichment analysis of FMGs. (**B**) KEGG enrichment analysis of FMGs. *ESCC* esophageal squamous cell carcinoma, *FMGs* fatty acid metabolism-related genes.

**Figure 4:**
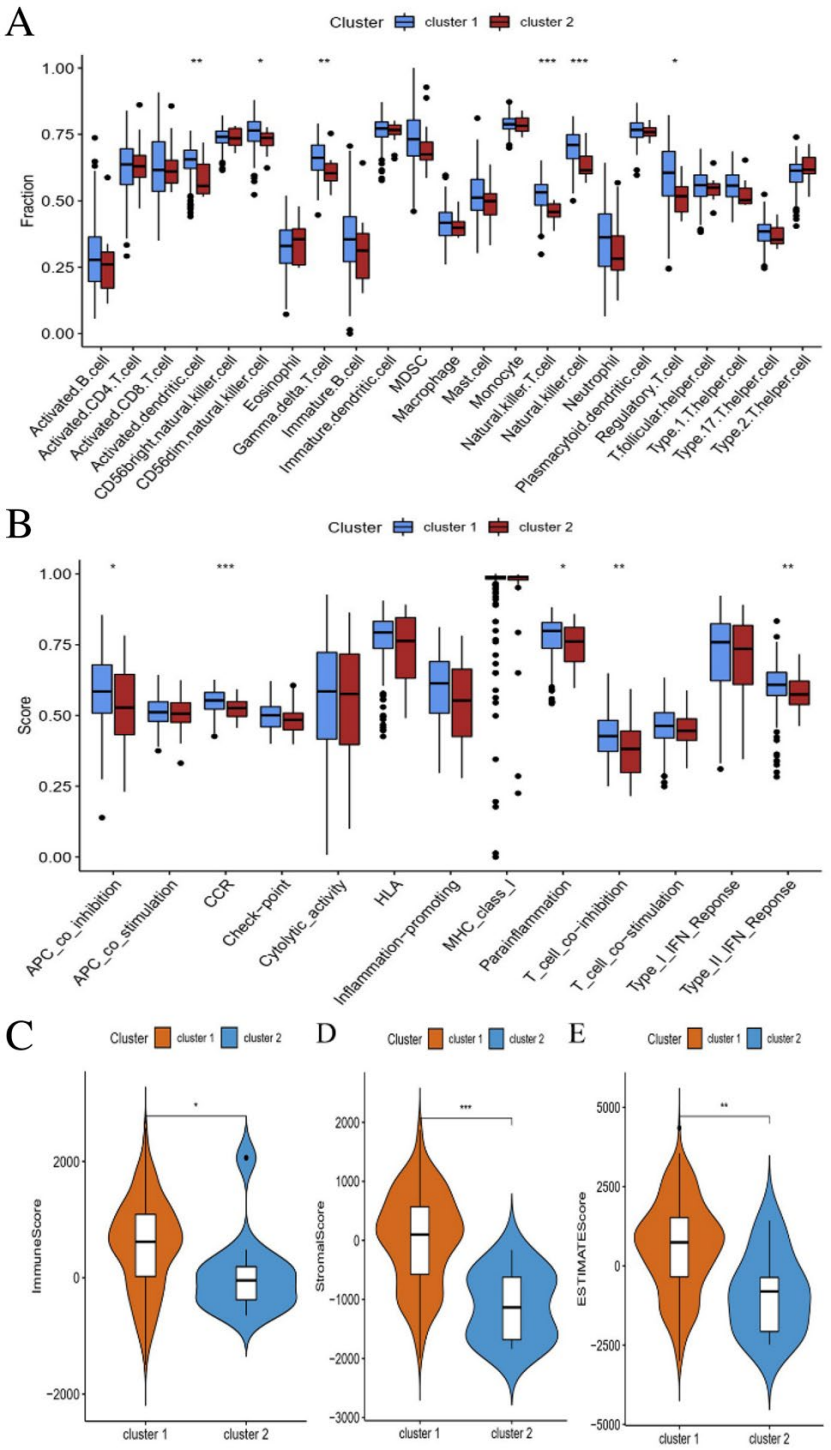
Analysis of the correlation between the ESCC subtypes and immune infiltration, immune functions and immune-related score. (**A**) Comparison of the ssGSEA scores among the two clusters. (**B**) The variation in immune functions. (**C**–**E**) The results of the correlation analysis between the immune-related score and subtypes. *p < 0.05; **p < 0.01; ***p < 0.001.

GO and KEGG functional analysis were conducted on the differentially expressed genes, with the enriched GO and KEGG terms in each category visualizing in Circos plots (Fig. 3A,B). The findings revealed that differentially expressed genes were remarkably associated with the FA metabolism related-regulation, such as FA degradation, arachidonic acid metabolism, FA elongation, beta-Alanine metabolism, and tryptophan metabolism (Table 2). The ssGSEA analysis revealed that the activated dendritic cells, CD56dim natural killer cells, gramma delta T cells, natural killer T cells, natural killer cells, and regulatory T cells had significantly higher in cluster 1 than in cluster 2 (Fig. 4A). Then, we analyzed the association with immune functions and found that APC co-inhibition, CCR, parainflammation, T cell co-inhibition, and type II IFN response had significantly different immune function scores in two subtypes (Fig. 4B). As shown in Fig. 4C–E, patients in cluster 1 had the higher stromal, immune score and ESTIMATE score than in cluster 2.

**Table 2:** Functional annotation of FMGs in ESCC.

Category	ID	Description	P-value
Biology process	GO:0006631	Fatty acid metabolic process	9.58E−90
	GO:0001676	Long-chain fatty acid metabolic process	9.07E−46
	GO:0033559	Unsaturated fatty acid metabolic process	3.46E−45
	GO:0006633	Fatty acid biosynthetic process	6.71E−45
	GO:0120254	Olefinic compound metabolic process	1.84E−42
	GO:0072330	Monocarboxylic acid biosynthetic process	6.60E−42
	GO:0005777	Peroxisome	6.32E−13
	GO:0042579	Microbody	6.32E−13
	GO:0005782	Peroxisomal matrix	1.54E−09
	GO:0031907	Microbody lumen	1.54E−09
	GO:0005759	Mitochondrial matrix	2.05E−08
	GO:0031301	Integral component of organelle membrane	1.08E−05
	GO:0016614	Oxidoreductase activity, acting on CH–OH group of donors	1.37E−19
	GO:0016616	Oxidoreductase activity, acting on the CH–OH group of donors, NAD or NADP as acceptor	1.75E−17
	GO:0016627	Oxidoreductase activity, acting on the CH–CH group of donors	2.36E−16
	GO:0016705	Oxidoreductase activity, acting on paired donors, with incorporation or reduction of molecular oxygen	3.94E−15
	GO:0005506	Iron ion binding	1.97E−13
	GO:0003995	Acyl-CoA dehydrogenase activity	5.81E−13
KEGG pathway	hsa00350	Tyrosine metabolism	1.72E−08
	hsa00380	Tryptophan metabolism	6.28E−08
	hsa04146	Peroxisome	1.13E−07
	hsa00410	Beta-alanine metabolism	1.25E−07
	hsa01212	Fatty acid metabolism	7.78E−37
	hsa00071	Fatty acid degradation	5.10E−31
	hsa00590	Arachidonic acid metabolism	1.64E−28
	hsa01040	Biosynthesis of unsaturated fatty acids	4.26E−22
	hsa03320	PPAR signaling pathway	2.15E−18
	hsa00280	Valine, leucine and isoleucine degradation	7.96E−16
	hsa00062	Fatty acid elongation	1.56E−14
	hsa00620	Pyruvate metabolism	6.07E−13
	hsa04936	Alcoholic liver disease	3.84E−12
	hsa00010	Glycolysis/gluconeogenesis	9.84E−10
	hsa00982	Drug metabolism—cytochrome P450	2.19E−09

*FMGs* Fatty acid metabolism-related genes.

### Construction of a risk model

The prognostic-related FMGs (*p* < 0.05) were screened using univariate Cox regression analysis and these genes were included into the LASSO regression model for screening the candidate risk model-related genes. A eight- FMGs risk model was finally established based on the appropriate λ value. The risk score calculating formula in the training cohort, validation cohort and all cohorts was as follows: risk score = (0.0422) × FZD10 + (0.0034) × TACSTD2 + (− 0.0941) × MUC4 + (0.0313) × PDLIM1 + (− 0.1334) × PRSS12 + (0.1114) × BAALC + (0.2159) × DNAJA2 + (− 0.0464) × ALOX12B.

### Clinical application of risk score in ESCC cohorts

**Figure 5:**
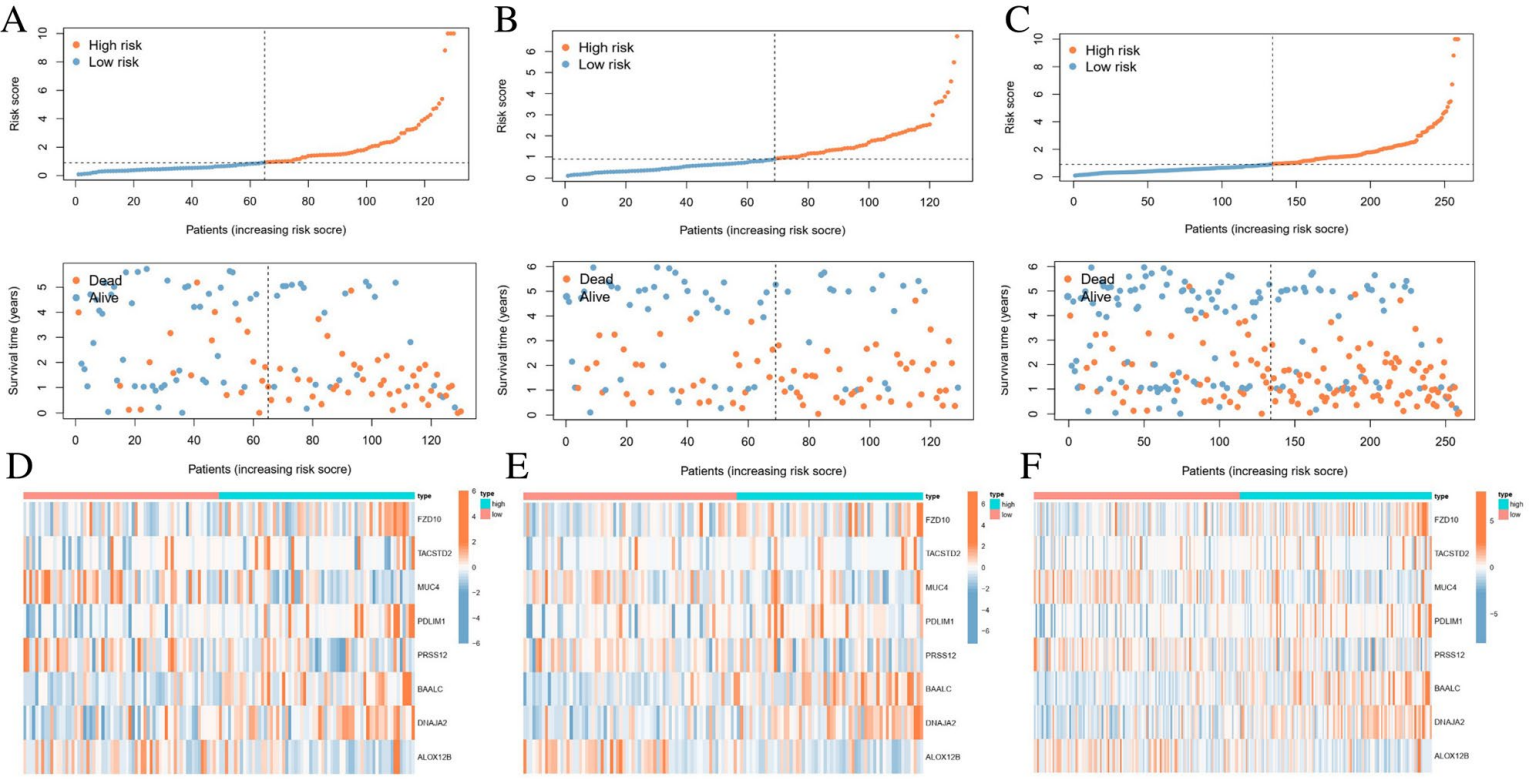
Construction of the prognostic risk model. The distribution plots of risk scores of ESCC patients in the (**A**) training, (**B**) validation, and (**C**) all cohorts. (**D**–**F**) Heatmap showing the expression profiles of the eight FMGs of ESCC patients in training, (**B**) validation, and (**C**) all cohorts. *ESCC* esophageal squamous cell carcinoma, *FMGs* fatty acid metabolism-related genes.

**Figure 6:**
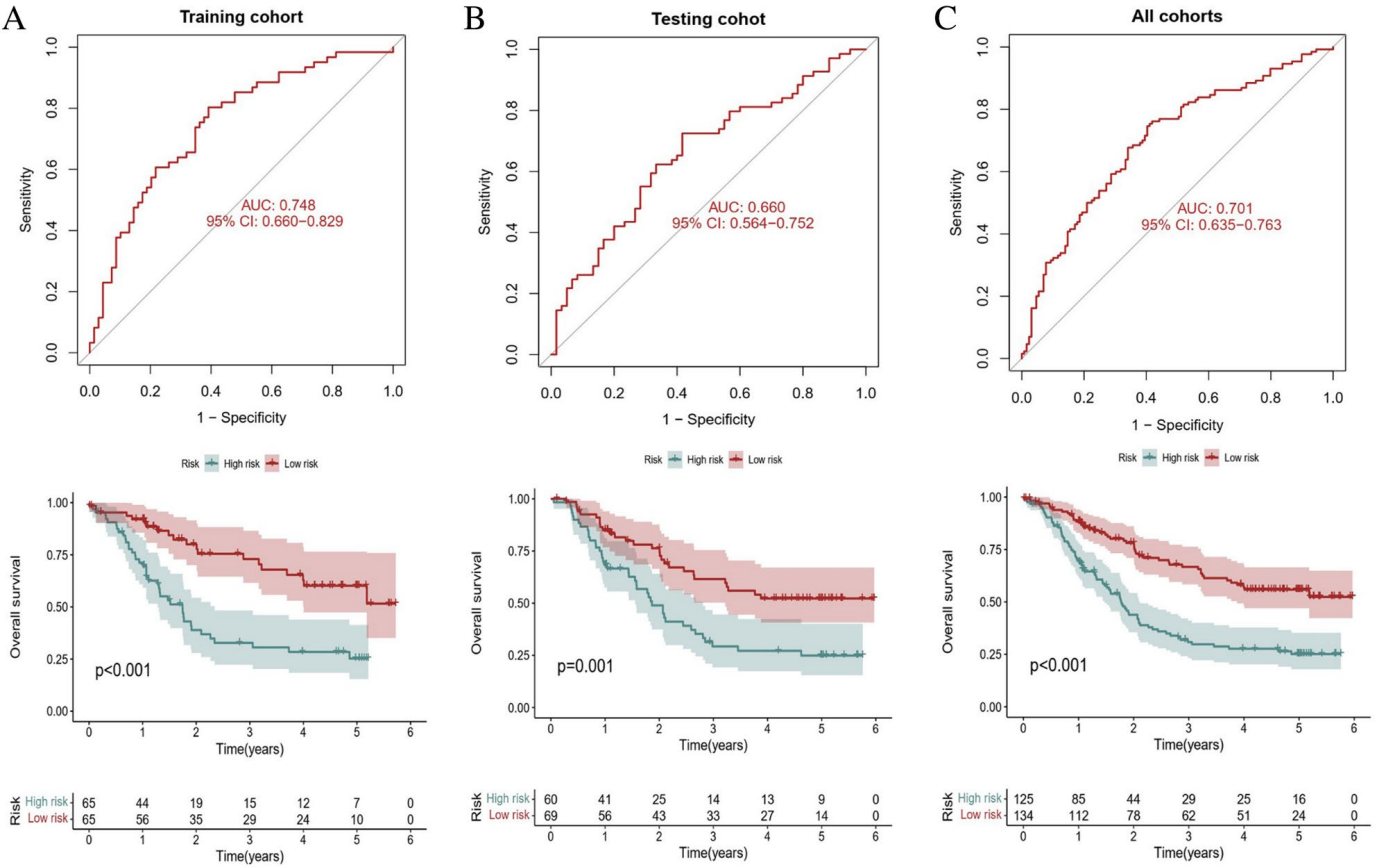
The Kaplan–Meier survival curves and ROC curves of risk score based on eight FMGs signature. Differences in the OS and predictive ability of risk model for ESCC patients between the high-risk and low-risk groups in the (**A**) training, (**B**) test, and (**C**) all cohorts. *ROC* receiver operator characteristic, *FMGs* fatty acid metabolism-related genes, *OS* overall survival, *ESCC* esophageal squamous cell carcinoma.

ESCC patients of training cohort, testing cohort and all cohorts were grouped into two risk groups depending on median value of risk scores. As shown in Fig. 5A–C, high-risk scores patients accounted for the higher percentage of patient deaths. A heatmap depicted the differential expression levels of genes among the different risk scores (Fig. 5D–F). The results of risk scores and survival rates in the training cohort were consistent with the testing cohort and all cohorts. The results revealed that high risk scores exhibited the unfavorable effect for prognosis. Kaplan–Meier survival analysis showed that patients in the low-risk survived longer than those in the high-risk group in the training cohort (*p* < 0.001), testing cohort (*p* = 0.001) and all cohorts (*p* < 0.001). The ROC curve results suggested that the AUCs for prognostic prediction were 0.748, 0.660, and 0.701, respectively (Fig. 6A–C).

### Construction of a prognosis nomogram

**Figure 7:**
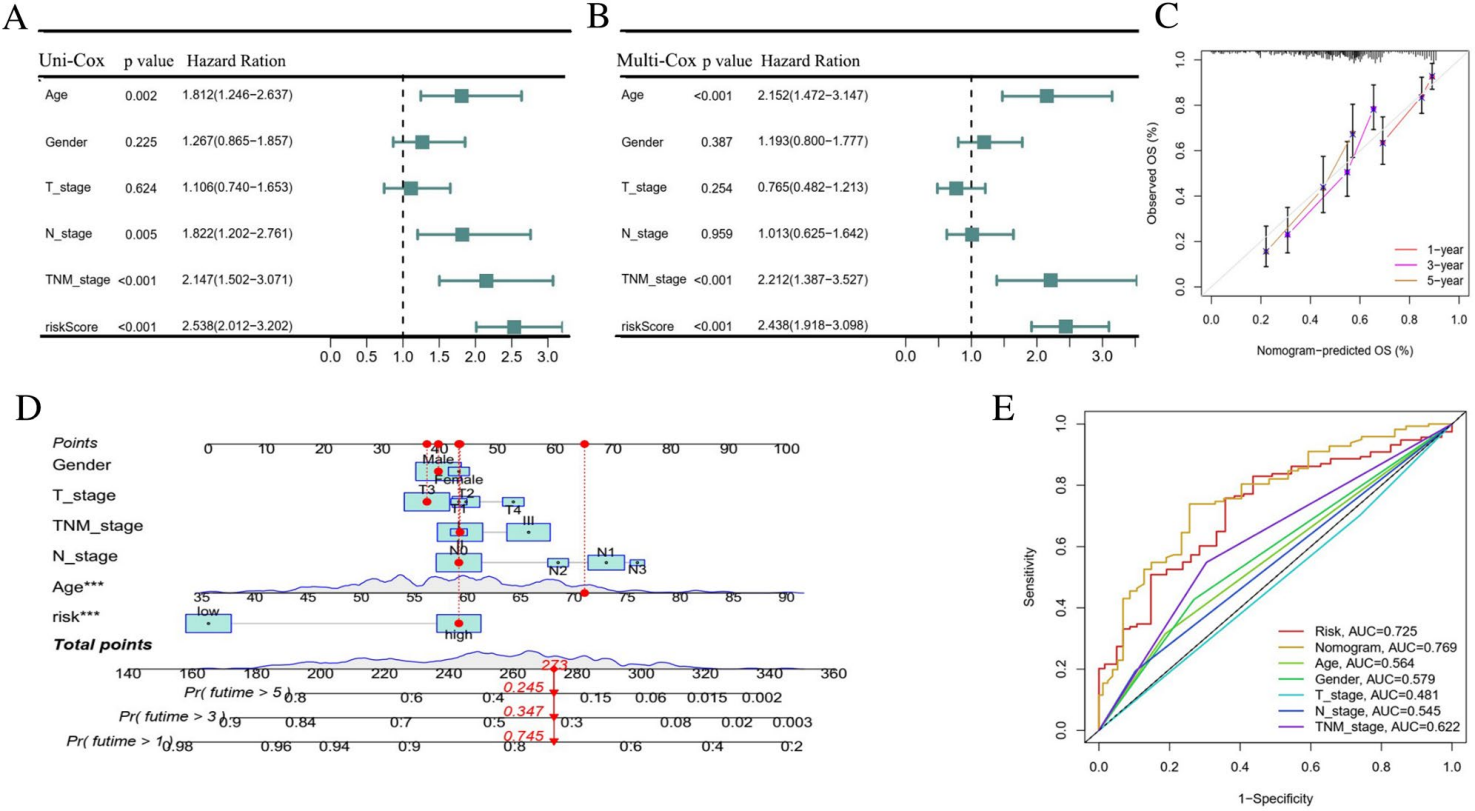
Independent prognostic validation of OS nomogram for ESCC patients. (**A**, **B**) Univariate and Multivariate analysis for all cohorts. (**C**, **D**) Nomogram and nomogram to predict 1-, 3-, and 5-year OS rates of ESCC patients. (**E**) ROC curves for clinical characteristics and nomogram. *OS* overall survival, *ESCC* esophageal squamous cell carcinoma, *ROC* receiver operator characteristic.

We performed the univariate and multivariate prognostic analyses to investigate the predictive ability of FA metabolism risk score for survival. Combining FA metabolism risk score with clinicopathological features in all cohorts, the results showed that the age (*p* < 0.001), TNM stage (*p* < 0.001), and risk score (*p* < 0.001) were proved as independent prognostic factors (Fig. 7A,B). Figure 7C showed calibration curves for predicting ESCC patients at OS of 1, 3, and 5 years, indicating that nomogram had reliably prediction capability. Considering the clear and visual characteristics of nomogram, so we visualized the risk signature using a clinicopathologic nomogram. A nomogram integrating age, gender, T stage, N stage, and risk score was analyzed to determine the relationship between these factors and prognosis (Fig. 7D). Further analysis indicated that the nomogram (AUC = 0.769) has a better predictive performance than a single prognostic indicator such age (AUC = 0.564), TNM stage (AUC = 0.622), or the prognostic risk scoring model (AUC = 0.725; Fig. 7E).

### Functional characteristics among two risk groups

**Figure 8:**
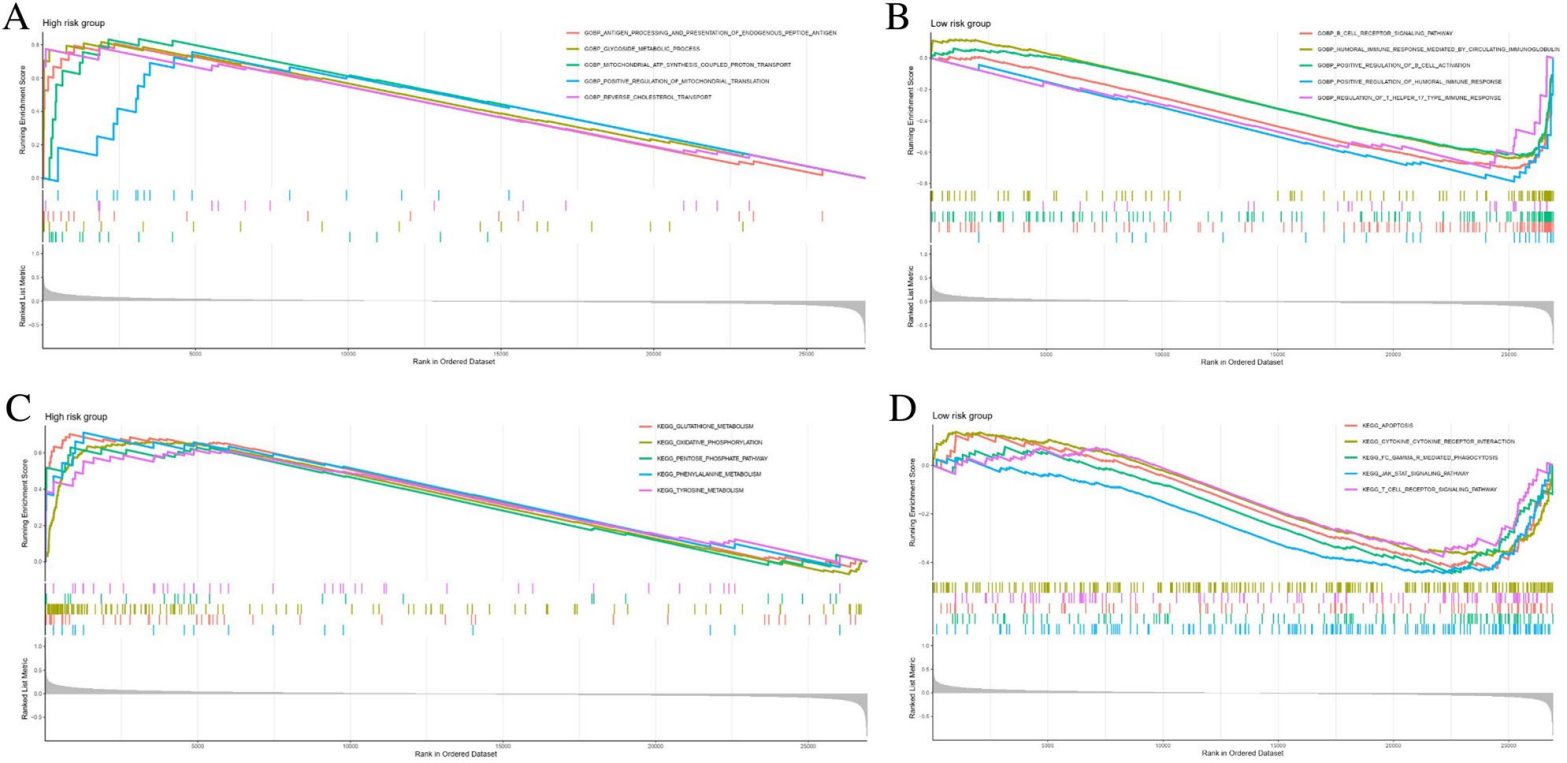
GSEA of the functional characteristics in the high-risk and low-risk groups. (**A**, **B**) GO function annotation among two risk groups. (**C**, **D**) The significantly enriched KEGG (https://www.kegg.jp/kegg/kegg1.html) pathways of different risk groups. *GSEA* gene set enrichment analysis, *GO* Gene Ontology, *KEGG* Kyoto Encyclopedia of Genes and Genomes, *ESCC* esophageal squamous cell carcinoma.

To further explore the potential molecular mechanism of the risk model, the transcript message in ESCC patients in the all cohorts were analyzed using GSEA. The GO analysis further revealed that glycoside metabolic process, reverse cholesterol transport, positive regulation of mitochondrial translation, mitochondrial ATP synthesis coupled proton transport, and antigen processing and presentation of endogenous peptide antigen were enriched in the high-risk group (Fig. 8A), and immune-related activities in low-risk group were shown in Fig. 8B. Interestingly, the activity of metabolic pathways such as phenylamine metabolism, glutathione metabolism, and tyrosine metabolism, were fundamentally enriched in the high-risk group, according to KEGG analysis (Fig. 8C). Additionally, JAK STAT signaling pathway, FC gamma R mediated phagocytosis, T cell receptor signaling pathway, cytokine-cytokine receptor interactions were enriched in the low-risk group (Fig. 8D). Taken together, our results revealed that the FA metabolism reprogramming condition can act on the immune activity through internal regulatory mechanisms.

### Risk score model to assess immune activity

**Figure 9:**
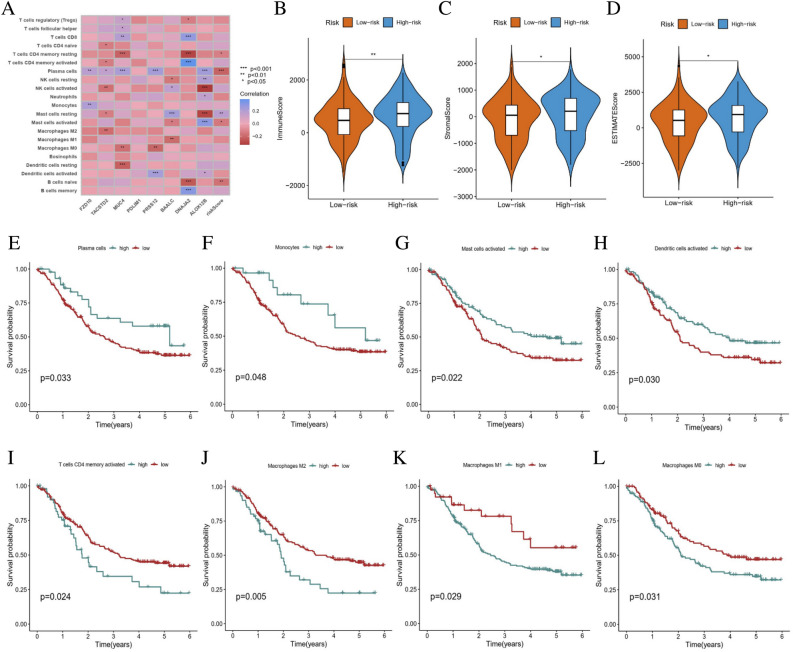
The correlation between the risk model and immune activity in ESCC patients. (**A**) The expression levels of immune cell infiltration in high-risk and low-risk groups. (**B**–**D**) Analysis of the association between the risk model and immune-related score. (**E**–**L**) Kaplan–Meier curves of OS in ESCC patients based on immune cells. *ESCC* esophageal squamous cell carcinoma; *p < 0.05; **p < 0.01; ***p < 0.001.

To examine the ESCC tumor microenvironment among the two risk groups patients, we explored the distribution of 21 immune cell types in different risk groups in all cohorts using CIBERSORT. The proportions of immune cells in the high-risk and low-risk groups were shown in Fig. 9A. The results indicated that the high-risk group had lower levels of naïve B cell, activated mast cells, plasma cells, and CD4 T cell memory resting, compared to the low-risk group. The StromalScore, ImmuneScore, and ESTIMATEScore were computed using the “ESTIMATE” package, revealing that ImmuneScore, StromalScore, and ESTIMATEScore were significantly higher in high-risk group than in low-risk group (*p* < 0*:*05) (Fig. 9B–D). Additionally, we evaluated the relationship between the tumor-infiltrating immune cells and prognosis, observing that increased plasma cells, monocytes, mast cells activated, and dendritic cells activated were closely related to good prognosis (Fig. 9E–H). High infiltration of T cells memory activated, M0 macrophages, M1 macrophages, and M2 macrophages showed unfavourable effects for survival (Fig. 9I–L).

**Figure 10:**
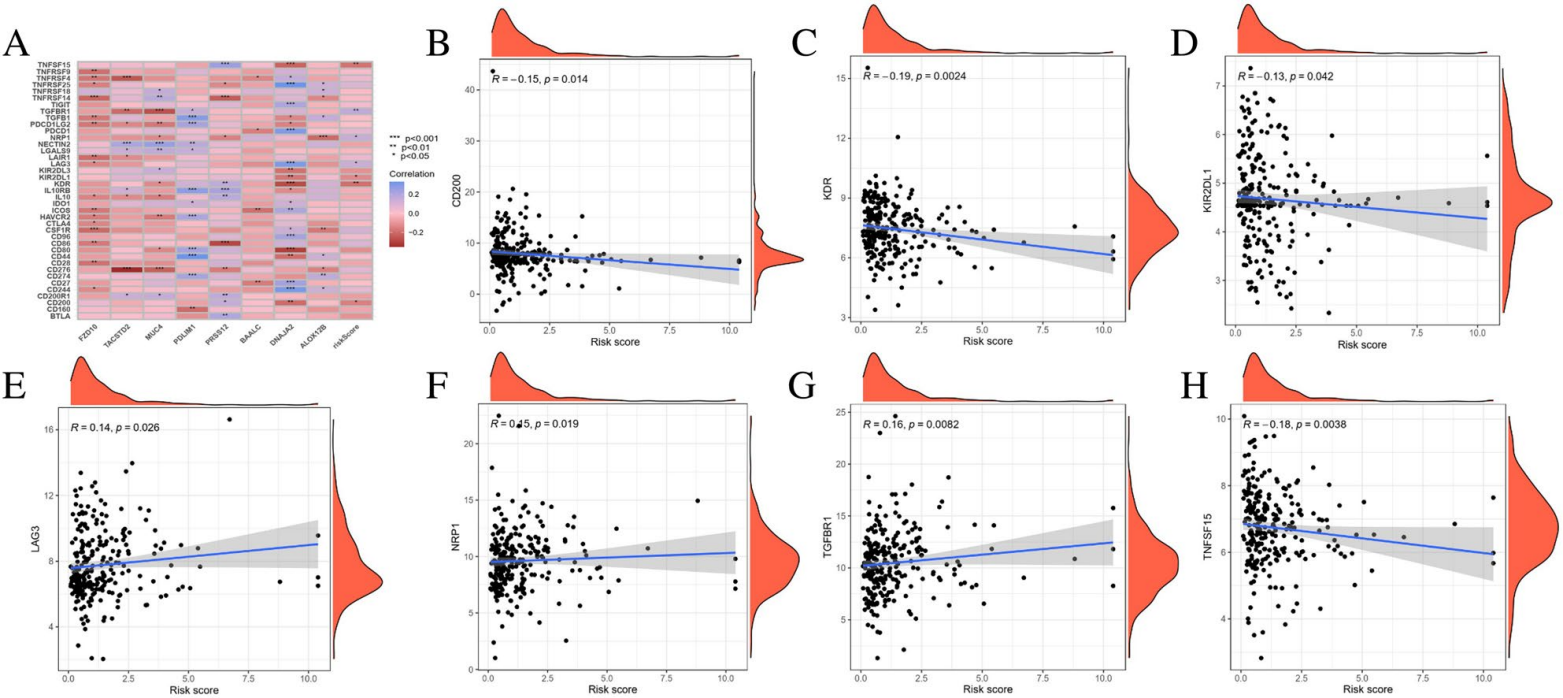
The correlation between the risk model and immune checkpoints in ESCC patients. (**A**) Heatmap of immune checkpoints with different risk score. (**B**–**H**) Correlation between the risk model and immune checkpoints. *ESCC* esophageal squamous cell carcinoma; *p < 0.05; **p < 0.01; ***p < 0.001.

The expression of 39 immune checkpoints in the high-risk and low-risk groups were also compared. The results revealed that most immune checkpoints were significantly expressed in different risk groups (Fig. 10A). Accordingly, the relationship between the immune checkpoints and risk score was performed, which suggested that the expression level of CD200, KDR, KIR2DL1, LAG3, NRP1, TGFBR1 and TNFSF15 were significantly associated with risk score (Fig. 10B–H).

### Validation of the expression levels of eight-FAMs signature in ESCC cells

**Figure 11:**
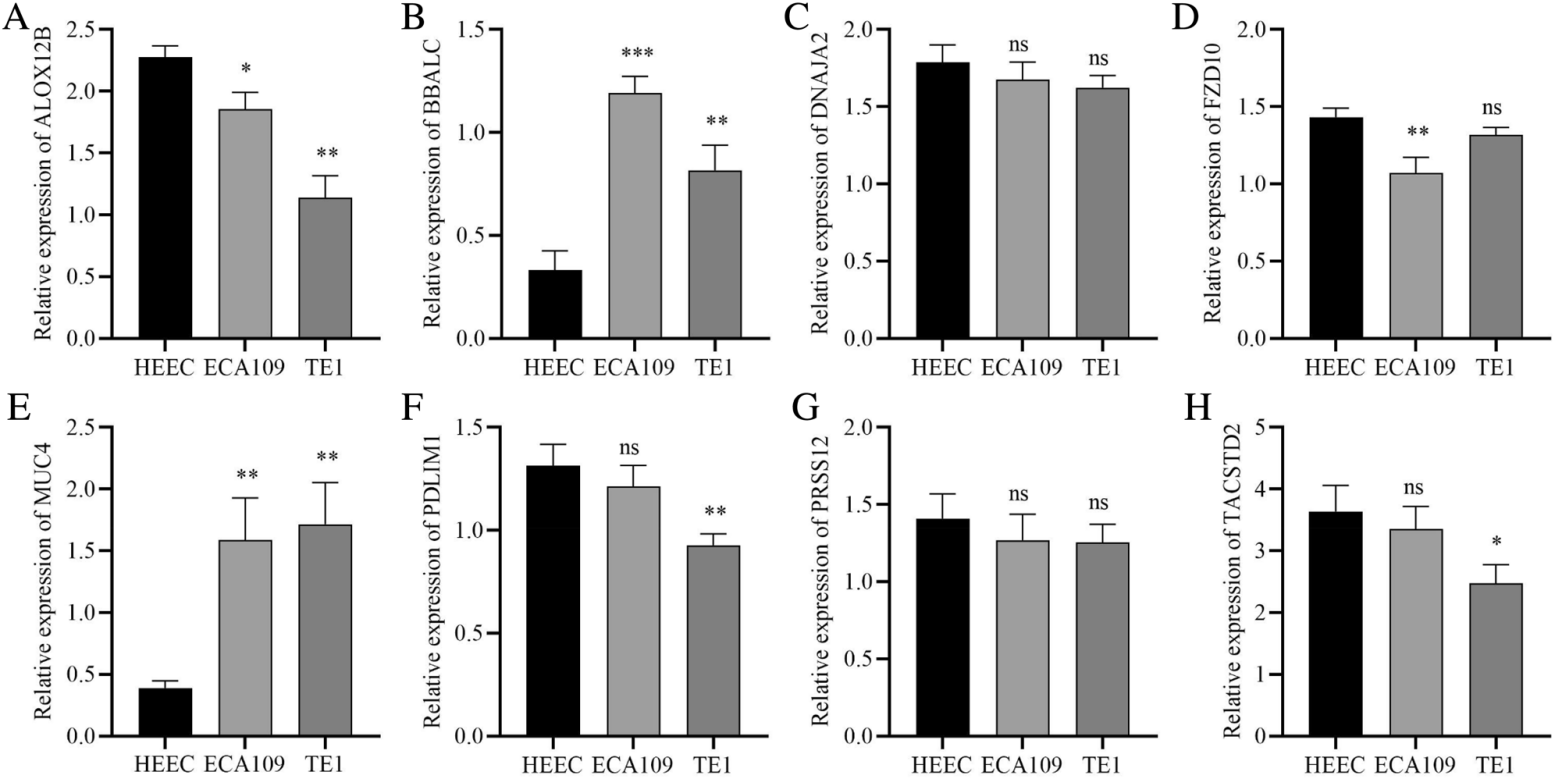
The expression levels of eight signature FMGs in ESCC cells. qRT-PCR analysis results of the expression levels of ALOX12B (**A**), BAALC (**B**), DNAJA2 (**C**), FZD10 (**D**), MUC4 (**E**), PDLIM1 (**F**), PRSS12 (**G**) and TACSTD2 (**H**) in HEEC, ECA109 and TE1. *qRT-PCR* quantitative real-time polymerase chain reaction, *FMGs* fatty acid metabolism-related genes, *ESCC* esophageal squamous cell carcinoma, *ns* not significant; *p < 0.05; **p < 0.01; ***p < 0.001.

The eight-FAMs risk model included the following genes: FZD10, TACSTD2, MUC4, PDLIM1, PRSS12, BAALC, DNAJA2 and ALOX12B. To further verify the expression level of the above mentioned FMGs, we detected the expression levels of these genes in HEEC, EAC109 and TE1 cells (Fig. 11A–H). The results of qRT-PCR revealed that the MUC4 and BAALC was upregulated, whereas the expression of TACSTD2, FZD10, PDLIM1, and ALOX12B was downregulated in ESCC cells than that in HEEC. However, we found no significant differences in expression levels of PRSS12 or DNAJA2 between HEEC and ESCC cell lines.

### Knockdown of MUC4 inhibited ESCC cell proliferation, migration and invasion

**Figure 12:**
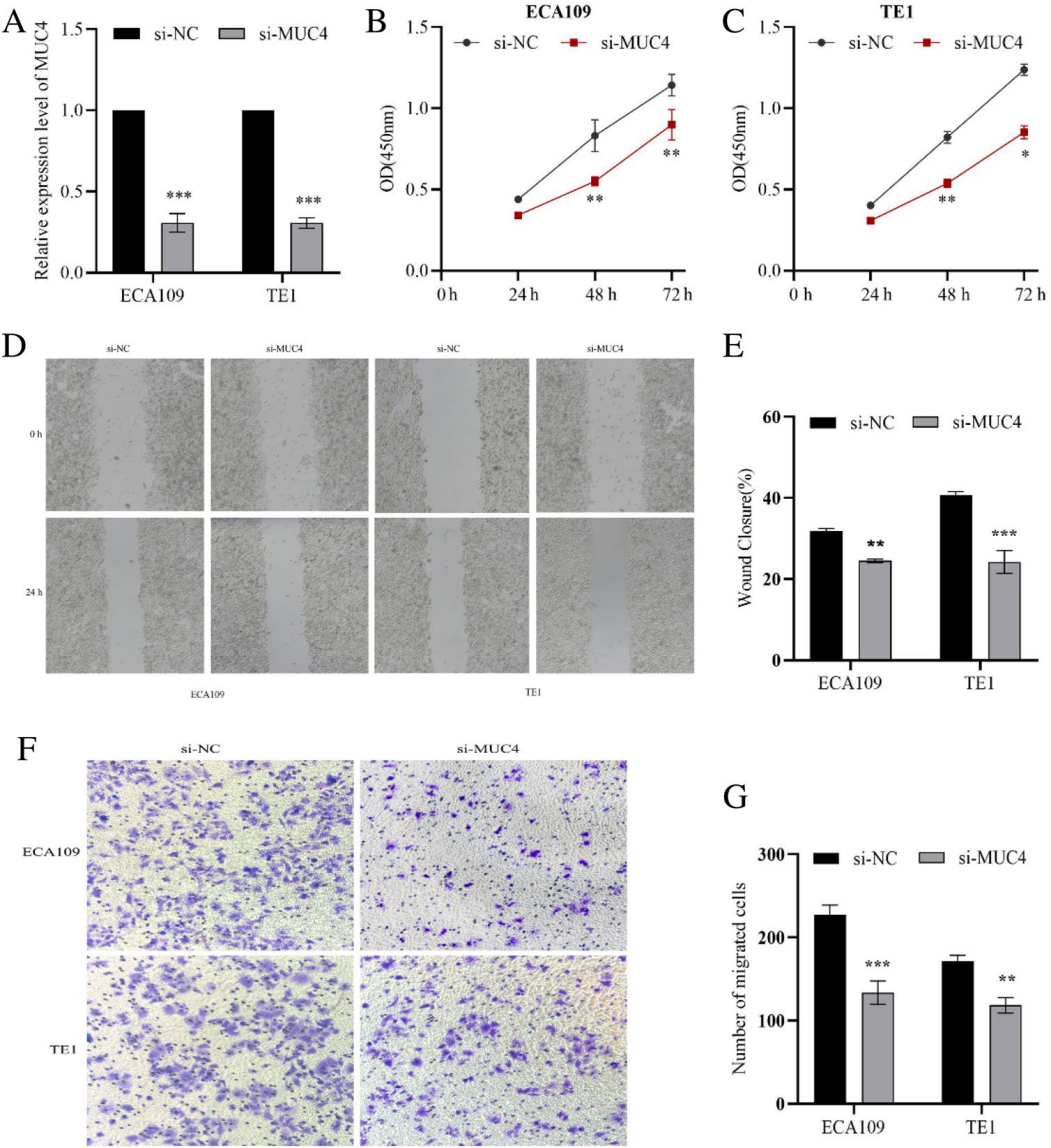
The functional roles of MUC4 for ECA109 and TE1 cells. (**A**) The transfection with si-MUC4 verified by qRT-PCR. (**B**, **C**) CCK-8 assay showed that knockdown of MUC4 resulted in growth retardation of ECA109 and TE1 cells. (**D**, **E**) Cell migration was evaluated with microscope (magnification, × 40). (**F**, **G**) The Transwell assay was used to detect the ability of cell invasion (magnification, × 100). *p < 0.05, **p < 0.01, and ***p < 0.001.

To explore the oncogenic roles of MUC4 in ESCC, we selected ECA109 and TE1 cells with low MUC4 expression by using si-MUC4 transfection subsequent experiments. MUC4 expression was knocked down in ECA109 and TE1 cells, and was demonstrated by qRT-PCR analysis (Fig. 12A). To evaluate the role of MUC4 on the proliferation, CCK-8 assay was used to validate and results showed the viability of MUC4 groups with knockdown were obviously lower than that of NC groups at 24, 48 and 72 h, respectively (Fig. 12B,C). Moreover, wound-healing assay suggested that after culture for 24 h, the knockdown of MUC4 expression significantly reduced the migration ability of ECA109 and TE1 cell lines (Fig. 12D,E). In addition, knockdown of MUC4 markedly attenuated the invasion ability in ECA109 and TE1 cell lines via transwell assay (Fig. 12F,G). Our findings showed that knockdown of MUC4 can suppresses ESCC cells proliferation, migration and invasion.

## Discussion

ESCC is a major public health problem in worldwide, which contributes to a poor prognosis because of the aggressiveness and treatment-resistant. Exploring corresponding biological mechanisms of ESCC progression can be beneficial for survival outcomes prediction. The rapid development of genomics and high-throughput sequencing could help to identify the prognostic-related genes, which has received great attention and is an urgent problem. Shi et al. built an epithelial-mesenchymal transition gene signature that could predict survival and immunotherapy efficacy^25^. Dai et al. constructed a prognostic signature of colorectal cancer based on a senescence phenotype-related clinical-molecular analysis. The results suggested that senescence-related genes signature could better predict the prognosis and facilitate individualized treatments^26^.

Li et al. found that DNA damage repair-related gene signature with cell cycle checkpoint function can predict the clinical outcomes and immune status of lung adenocarcinoma patients^27^. Cellular metabolism is of pivotal contribution to cell proliferation, angiogenesis, proliferation, and invasion, and dysregulated metabolism in cancer cells occurs due to the imbalance of tumor suppressor and oncogenic genes^28–30^. And dysregulated metabolism is widely observed in cancer cells and is used as target to improve cancer therapeutics^31–33^. More and more evidences indicated the increased levels of fatty acylcarnitines is one of the physiological features of cancer cells and supports energy production, which suggested that disturbances in fatty acid metabolism played an important role in tumor progression^34^. Moreover, FA metabolism influences remarkably metabolic-related signaling pathways for further altering the cancer cell biology by the synthesis of lipid building blocks for membranes^35^. Studies have shown that the characteristics of FA metabolism of patients with lung adenocarcinoma^36^, glioma^37^ and colorectal cancer^38^ may be used to guide clinical treatment. Previous studies have reported that FA metabolism-related signature associated with breast cancer patients’ prognosis^39^. Nevertheless, FA metabolism not only acts as a pivotal prognostic distinguishing index indicator, but also associates with cell morphology and cell function, which involves FA metabolism-related genes changes. To date, no FA metabolism-related gene signature and risk model have been constructed to predict long-term patient survival and immune activity by exploring FMGs in ESCC. Herein, a comprehensive study of characteristics of FA metabolism in ESCC is urgent.

In the present study, we analyzed publicly available databases transcriptome data for screening FMGs and identified prognostic-related genes, aiming to filter out genes associated with FA metabolism. Then, ESCC patients were divided into two cluster subtypes (cluster 1, cluster 2) based on these FMGs by consensus clustering analysis. We found that patients in cluster 2 had poorer survival outcomes that in cluster 1. Tumorigenic pathways (PPAR signaling pathway, glycolysis, drug metabolism-cytochrome P450, monocarboxylic acid biosynthetic process, etc.) were remarkably activated in cluster 2 subtype, which suggested unfavorable risk factors for patients in cluster 2. Cluster 1 and cluster 2 differed remarkably in tumor-infiltrating immune cells, including the activated dendritic cells, CD56 dim natural killer cells, gramma delta T cells, natural killer T cells, natural killer cells, and regulatory T cells. Cluster 1 had the higher stromal, immune score and ESTIMATE score than that in cluster 2. Therefore, we speculated that molecular subtypes of FMGs were associated with ESCC progression and immune response.

Additionally, we constructed a prognostic risk model based on eight FMGs for ESCC patients in the training cohort, validation cohort and all cohorts. The results indicated that the eight FMGs signature may have a cross-platform feature with favourable predictive accuracy for ESCC survival and generalizability in clinical practice. We divided the ESCC patients into high-risk and low-risk groups based on the cut-off values of the risk scores, and two risk groups suggested the different levels of FA metabolism. The Kaplan–Meier survival analysis indicated that low-risk group patients exhibited a better survival than that in high-risk group patients. This prognostic risk model exhibited a considerable accuracy in prognosis prediction than other features. The validation cohort and all cohorts yielded the consistent results, suggesting that the prognostic risk model may identify patients with different risk exposure. The Univariate and multivariate Cox regression analyses showed that the FA metabolism risk score was an independent prognostic factor for ESCC patients. Importantly, a nomogram consisting of age, gender, T stage, N stage and FA metabolism risk score was constructed and yielded a favorable predictive performance.

The prognostic risk model in present study was developed, which combined eight FMGs displayed a favorable survival prediction value in ESCC. Several studies have revealed that FZD10 is associated with the activation of Wnt signalling in colorectal cancers^40^ and gastric cancers^41^, and enhances their anchorage-independent growth and induces epithelial-mesenchymal transition (EMT) in breast cancer cells^42^. TACSTD2 (Trophoblast Cell Surface Antigen 2) has been reported to be associated with malignant tumors. Shvartsur et al. reported that TACSTD2 promoted tumor cells EMT, adhesion and proliferation, and stimulate the formation of tumors^43^. Ambrogi et al. analyzed the expression level of TACSTD2 using immunohistochemistry and observed that high TACSTD2 expression was a negative prognostic factor^44^. The low expression of MUC4 was associated with favorable prognosis and survival^45^. In addition, studies have shown that MUC4 can interact with key tumor-related signaling pathways to promote tumor cells proliferation, invasion, metastasis, and chemoresistance in pancreatic cancer^46^. PDLIM1 (PDZ and LIM domain protein 1) plays an important role in the invasion, EMT, metastasis, and progression of colorectal cancer, suggesting that PDLIM1 is a potential therapeutic target for diagnosis and treatment of tumors in the clinic^47^. The knockdown of BAALC expression reduced the abilities of proliferation, invasion and migration for breast cancer cells^48^. ALOX12B serves significant roles in the carcinogenesis in cervical cancer by the PI3K/ERK1 signaling pathway^49^, and ALOX12B can inhibit immune activity and increased risk of lung cancer^50^. In study, the associations between ESCC and FZD10, TACSTD2, PDLIM1, PRSS12, DNAJA2, and ALOX12B were first to report. Nevertheless, whether these risk model-related FMGs influence metabolism and generate the prognostic values in patients with ESCC, remains ambiguous owing to the limited number of studies.

ESCC serving as an immunogenic tumor, we also actively explore the critical features of immune activity in ESCC. Numerous studies have reported that FA metabolism induces the changes in the proportion of infiltrating immune cells in tumor immune microenvironment^51–53^. For this purpose, we analyzed the immune infiltration status of 21 immune cells among different risk groups, revealing that immune cells, including naïve B cell, activated mast cells, plasma cells, and CD4 T cell memory resting were increased in the low-risk group than that in high-risk group. Subsequently, we found that the expression of LAG3, NRP1, and TGFBR1 increased considerably with increasing risk scores. These findings suggested that patients with increasing risk score may benefit from precision immunotherapies that target LAG3, TNFSF15, KIR2DL1, KDR, CD200, NRP1, and TGFBR1. From this, we speculated that this prognostic risk model may provide valuable clues of ESCC patients for immunotherapy, further confirming that FA metabolism is indispensable in remodeling the tumor immune microenvironment.

Some limitations of present study must be considered. First, the molecular subtypes and risk model were constructed using TCGA and GEO databases. Larger and multicenter clinical samples are required to validate the performance of the molecular subtypes and risk model in ESCC patients. Second, the FMGs signature screened by ESCC samples have not been previously analyzed, further study into their underlying mechanisms for judging the prognosis is required. Third, although we have preliminarily investigated the effects of FMGs signature on immune activity, in vivo and vitro experiments should be conducted to further validate the prognostic performance of our proposed FMGs signature for ESCC. Fourth, only MUC4 expression was knocked down in proliferation assay, wound-healing assay and cell invasion assay. We will conduct a series of in vivo and vitro assays on other risk model-related genes.

## Conclusion

Our study fills the gap of FMGs signature for in predicting prognosis and immune activity of ESCC. These findings could provide a more detailed portrait of FMGs in the biology features of ESCC, which facilities individualized immunotherapeutic options to improve prognoses.

## Data Availability

Data is provided within the manuscript or available from the corresponding author on reasonable request.
